# (*Z*)-3-Ferrocenyl-2-(3-pyrid­yl)­acrylo­nitrile

**DOI:** 10.1107/S1600536808025531

**Published:** 2008-08-16

**Authors:** Fang Chen, Heng-Yun Ye

**Affiliations:** a Ordered Matter Science Research Center, College of Chemistry and Chemical Engineering, Southeast University, Nanjing 210096, People’s Republic of China

## Abstract

The mol­ecular structure of the title compound, [Fe(C_5_H_5_)(C_13_H_9_N_2_)], (I), is analogous to that of the compound (*Z*)-3-ferrocenyl-2-phenyl­acrylonitrile [Cao & Ye (2008). *Acta Cryst.* E**64**, m822], (II), with the pyridine ring in (I) replacing the benzene ring in (II). While the corresponding bond distances and angles in the two compounds show no significant differences, the two dihedral angles between the planes through the acrylonitrile group and the two rings attached to it (substituted Cp and pyridine) of 16.8 (4) and 20.1 (4)° in (I) are different from the corresponding dihedral angles [19.6 (3) and 6.5 (4)°] in (II). The unsubstituted ring is disordered over two positions, with site-occupancy factors of 0.70 (1) and 0.30 (1). The major and minor components of the disordered ring are almost coplanar and are also parallel to the substituted cyclo­penta­diene ring plane, with a dihedral angle of 0.3 (6)°.

## Related literature

For background to the chemistry of ferrocene, see: Long (1995 ▶); Roberto *et al.* (2000 ▶); Togni & Hayashi (1995 ▶). For the structure of an analogous compound, see: Cao & Ye (2008 ▶). For bond-length data, see: Allen *et al.* (1987 ▶).
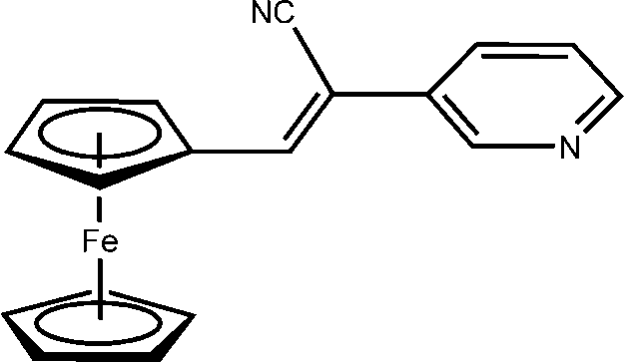


## Experimental

### 

#### Crystal data

[Fe(C_5_H_5_)(C_13_H_9_N_2_)]
*M* *_r_* = 314.16
Monoclinic, 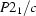

*a* = 11.552 (3) Å
*b* = 9.2557 (15) Å
*c* = 14.458 (5) Å
β = 111.679 (15)°
*V* = 1436.6 (7) Å^3^
*Z* = 4
Mo *K*α radiation
μ = 1.04 mm^−1^
*T* = 293 (2) K
0.25 × 0.2 × 0.1 mm

#### Data collection

Rigaku SCXmini diffractometer
Absorption correction: multi-scan (*CrystalClear*; Rigaku, 2005 ▶) *T*_min_ = 0.896, *T*_max_ = 1.000 (expected range = 0.808–0.901)
14447 measured reflections
3290 independent reflections
2008 reflections with *I* > 2σ(*I*)
*R*_int_ = 0.084

#### Refinement

*R*[*F* ^2^ > 2σ(*F* ^2^)] = 0.053
*wR*(*F* ^2^) = 0.107
*S* = 0.99
3290 reflections
206 parameters
22 restraints
H-atom parameters constrained
Δρ_max_ = 0.24 e Å^−3^
Δρ_min_ = −0.27 e Å^−3^

### 

Data collection: *CrystalClear* (Rigaku, 2005 ▶); cell refinement: *CrystalClear*; data reduction: *CrystalClear*; program(s) used to solve structure: *SHELXS97* (Sheldrick, 2008 ▶); program(s) used to refine structure: *SHELXL97* (Sheldrick, 2008 ▶); molecular graphics: *SHELXTL* (Sheldrick, 2008 ▶); software used to prepare material for publication: *SHELXL97*.

## Supplementary Material

Crystal structure: contains datablocks I, global. DOI: 10.1107/S1600536808025531/ez2133sup1.cif

Structure factors: contains datablocks I. DOI: 10.1107/S1600536808025531/ez2133Isup2.hkl

Additional supplementary materials:  crystallographic information; 3D view; checkCIF report

## Figures and Tables

**Fig. 1.**
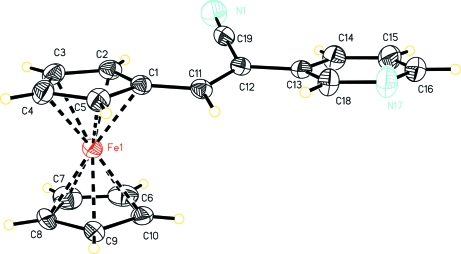
The structure of I showing the atom numbering scheme with displacement ellipsoids drawn at the 30% probability level. For clarity only atoms of the major disorder component of the unsubstituted cyclopentadiene ring are included.

## supplementary crystallographic information

### Comment

The chemistry of ferrocene has received much attention because of its applications in many fields, such as in catalysis, organic or organometallic synthesis and materials design (Togni & Hayashi, 1995). The use of ferrocene and its derivatives as non-linear optical (NLO) materials has also been reported (Long, 1995; Roberto *et al.*, 2000). As part of our on-going studies into the chemistry of ferrocene, we report the crystal structure of the title compound (I), Fig. 1.

I is analogous to the compound (*Z*)-2-Phenyl-3-(ferrocenyl)acrylonitrile (II) (Cao & Ye, 2008), except for the replacement of the benzene ring in II by the pyridine ring in I. The two molecular structures show no significant differences between the corresponding bond distances and angles. Although the two compounds have the same spacegroup (P 2_1_/c) their crystal structures are obviously different, since the unit cell parameters differ. This is because the two dihedral angles between the planes through the acrylonitrile group and the two attached rings (the substituted Cp ring, C1–C5, and the pyridine ring, C13–C18) [16.8 (4)°, 20.1 (4)°] in I are different from the corresponding dihedral angles [6(2)°, 6.5 (4)°] in II. The unsubstituted Cp ring is disordered over two positions, with site occupancy factors 0.70 (1) and 0.30 (1). The major (C6–C10) and the minor (C6'–C10') components of the disordered ring are almost coplanar with mean deviations of 0.0239 Å from the planes. Both disordered rings are parallel to the C1–C5 ring plane with dihedral angles of 0.3 (6)°. The major component is in an eclipsed configuration relative to the substituted Cp ring with a C1-Cg1-Cg2-C10 torsion angle of -0.2 (4)°; while the minor component is staggered, with a C1-Cg1-Cg2-C10' torsion angle of 24 (2)° [Cg(1) denotes the centroid of the substituted Cp ligand; Cg(2) denotes the centroid of the unsubstituted Cp ligand]. The iron-ring centroid distances are Fe—Cg(1), 1.638 (2) Å; Fe—Cg(2), 1.6282 (5) Å. Within the acrylonitrile unit, bond distances and angles are normal (Allen *et al.*, 1987).

### Experimental

The title compound was prepared by an analogous procedure to that for (*Z*)-2-Phenyl-3-(ferrocenyl)acrylonitrile (Cao & Ye, 2008). Red crystals suitable for X-ray analysis were obtained by slow evaporation of a saturated ethyl ether solution.

### Refinement

All H-atoms were positioned geometrically and refined using a riding model with d(C-H) = 0.93Å, U_iso_=1.2U_eq_ (C). The unsubstituted Cp ring is disordered over two positions with site occupancy factors 0.70 (1) and 0.30 (1) respectively; corresponding C atoms were restrained to have the same anisotropic displacement parameters.

### Crystal data

**Table:**

[Fe(C_5_H_5_)(C_13_H_9_N_2_)]	*F*_000_ = 648
*M**_r_* = 314.16	*D*_x_ = 1.453 Mg m^−^^3^
Monoclinic, *P*2_1_/*c*	Mo *K*α radiation λ = 0.71073 Å
Hall symbol: -P 2ybc	Cell parameters from 2602 reflections
*a* = 11.552 (3) Å	θ = 2.7–27.5º
*b* = 9.2557 (15) Å	µ = 1.04 mm^−^^1^
*c* = 14.458 (5) Å	*T* = 293 (2) K
β = 111.679 (15)º	Block, red
*V* = 1436.6 (7) Å^3^	0.25 × 0.2 × 0.1 mm
*Z* = 4	

### Data collection

**Table:**

Rigaku SCXmini diffractometer	3290 independent reflections
Radiation source: fine-focus sealed tube	2008 reflections with *I* > 2σ(*I*)
Monochromator: graphite	*R*_int_ = 0.084
Detector resolution: 13.6612 pixels mm^-1^	θ_max_ = 27.5º
*T* = 293(2) K	θ_min_ = 2.7º
ω scans	*h* = −14→14
Absorption correction: Multi-scan(CrystalClear; Rigaku, 2005)	*k* = −12→12
*T*_min_ = 0.896, *T*_max_ = 1.000	*l* = −18→18
14447 measured reflections	

### Refinement

**Table:**

Refinement on *F*^2^	Secondary atom site location: difference Fourier map
Least-squares matrix: full	Hydrogen site location: inferred from neighbouring sites
*R*[*F*^2^ > 2σ(*F*^2^)] = 0.053	H-atom parameters constrained
*wR*(*F*^2^) = 0.107	*w* = 1/[σ^2^(*F*_o_^2^) + (0.0415*P*)^2^] where *P* = (*F*_o_^2^ + 2*F*_c_^2^)/3
*S* = 0.99	(Δ/σ)_max_ = 0.006
3290 reflections	Δρ_max_ = 0.24 e Å^−^^3^
206 parameters	Δρ_min_ = −0.27 e Å^−^^3^
22 restraints	Extinction correction: none
Primary atom site location: structure-invariant direct methods	

### Special details

**Table:**

Geometry. All esds (except the esd in the dihedral angle between two l.s. planes) are estimated using the full covariance matrix. The cell esds are taken into account individually in the estimation of esds in distances, angles and torsion angles; correlations between esds in cell parameters are only used when they are defined by crystal symmetry. An approximate (isotropic) treatment of cell esds is used for estimating esds involving l.s. planes.
Refinement. Refinement of F^2^ against ALL reflections. The weighted R-factor wR and goodness of fit S are based on F^2^, conventional R-factors R are based on F, with F set to zero for negative F^2^. The threshold expression of F^2^ > 2sigma(F^2^) is used only for calculating R-factors(gt) etc. and is not relevant to the choice of reflections for refinement. R-factors based on F^2^ are statistically about twice as large as those based on F, and R- factors based on ALL data will be even larger.

### Fractional atomic coordinates and isotropic or equivalent isotropic displacement parameters (Å^2^)

**Table:**

	*x*	*y*	*z*	*U*_iso_*/*U*_eq_	Occ. (<1)
Fe1	0.23682 (4)	0.84541 (5)	0.05742 (3)	0.04190 (16)	
C1	0.2053 (3)	0.6292 (3)	0.0517 (2)	0.0394 (8)	
C2	0.3166 (3)	0.6649 (4)	0.1352 (2)	0.0467 (8)	
H2	0.3963	0.6306	0.1463	0.056*	
C3	0.2840 (3)	0.7601 (4)	0.1970 (3)	0.0546 (9)	
H3	0.3391	0.7994	0.2560	0.065*	
C4	0.1550 (4)	0.7868 (4)	0.1557 (3)	0.0541 (9)	
H4	0.1103	0.8459	0.1826	0.065*	
C5	0.1059 (3)	0.7077 (3)	0.0662 (3)	0.0470 (8)	
H5	0.0227	0.7064	0.0236	0.056*	
C6	0.3482 (8)	0.9264 (10)	−0.0106 (9)	0.071 (2)	0.699 (7)
H6	0.4200	0.8829	−0.0120	0.085*	0.699 (7)
C7	0.3419 (10)	1.0228 (12)	0.0626 (9)	0.077 (2)	0.699 (7)
H7	0.4092	1.0545	0.1174	0.092*	0.699 (7)
C8	0.2160 (10)	1.0636 (7)	0.0390 (7)	0.0603 (19)	0.699 (7)
H8	0.1857	1.1252	0.0756	0.072*	0.699 (7)
C9	0.1453 (6)	0.9930 (9)	−0.0507 (6)	0.0509 (16)	0.699 (7)
H9	0.0597	1.0014	−0.0840	0.061*	0.699 (7)
C10	0.2262 (10)	0.9071 (8)	−0.0817 (5)	0.0542 (18)	0.699 (7)
H10	0.2033	0.8490	−0.1381	0.065*	0.699 (7)
C10'	0.303 (3)	0.904 (2)	−0.0488 (16)	0.0542 (18)	0.301 (7)
H10'	0.3310	0.8412	−0.0860	0.065*	0.301 (7)
C9'	0.1810 (19)	0.950 (2)	−0.0742 (13)	0.0509 (16)	0.301 (7)
H9'	0.1135	0.9227	−0.1304	0.061*	0.301 (7)
C8'	0.178 (2)	1.039 (2)	−0.0049 (18)	0.0603 (19)	0.301 (7)
H8'	0.1062	1.0863	−0.0065	0.072*	0.301 (7)
C7'	0.298 (3)	1.056 (3)	0.075 (2)	0.077 (2)	0.301 (7)
H7'	0.3194	1.1108	0.1326	0.092*	0.301 (7)
C6'	0.377 (2)	0.967 (3)	0.041 (2)	0.071 (2)	0.301 (7)
H6'	0.4620	0.9543	0.0730	0.085*	0.301 (7)
C11	0.1881 (3)	0.5403 (3)	−0.0341 (2)	0.0403 (8)	
H11A	0.1145	0.5558	−0.0882	0.048*	
C12	0.2634 (3)	0.4379 (3)	−0.0470 (2)	0.0381 (7)	
C13	0.2356 (3)	0.3444 (3)	−0.1358 (2)	0.0396 (7)	
C14	0.3280 (3)	0.2680 (4)	−0.1533 (3)	0.0576 (10)	
H14A	0.4108	0.2790	−0.1112	0.069*	
C15	0.2968 (4)	0.1755 (4)	−0.2333 (3)	0.0660 (11)	
H15A	0.3581	0.1237	−0.2463	0.079*	
C16	0.1742 (4)	0.1610 (4)	−0.2935 (3)	0.0642 (11)	
H16A	0.1539	0.0978	−0.3471	0.077*	
C18	0.1149 (3)	0.3232 (4)	−0.2021 (3)	0.0547 (9)	
H18A	0.0517	0.3756	−0.1922	0.066*	
C19	0.3819 (3)	0.4094 (4)	0.0320 (3)	0.0484 (9)	
N1	0.4752 (3)	0.3827 (4)	0.0921 (2)	0.0740 (10)	
N17	0.0830 (3)	0.2324 (4)	−0.2791 (2)	0.0676 (9)	

### Atomic displacement parameters (Å^2^)

**Table:**

	*U*^11^	*U*^22^	*U*^33^	*U*^12^	*U*^13^	*U*^23^
Fe1	0.0474 (3)	0.0360 (3)	0.0422 (3)	−0.0029 (2)	0.0163 (2)	0.0004 (2)
C1	0.0403 (18)	0.0351 (19)	0.0415 (18)	−0.0043 (14)	0.0137 (16)	0.0013 (14)
C2	0.0431 (19)	0.045 (2)	0.0422 (18)	−0.0018 (16)	0.0043 (16)	0.0055 (16)
C3	0.069 (3)	0.053 (2)	0.0353 (18)	−0.010 (2)	0.0118 (19)	0.0001 (17)
C4	0.066 (3)	0.056 (2)	0.048 (2)	−0.0045 (19)	0.029 (2)	−0.0031 (17)
C5	0.045 (2)	0.049 (2)	0.048 (2)	−0.0048 (16)	0.0181 (17)	−0.0001 (16)
C6	0.060 (4)	0.069 (6)	0.089 (7)	−0.006 (4)	0.033 (4)	0.026 (5)
C7	0.082 (7)	0.056 (7)	0.079 (5)	−0.027 (5)	0.015 (5)	0.003 (3)
C8	0.096 (6)	0.028 (3)	0.052 (5)	0.002 (3)	0.021 (4)	−0.007 (3)
C9	0.065 (4)	0.041 (4)	0.050 (4)	0.014 (3)	0.024 (3)	0.004 (3)
C10	0.074 (5)	0.049 (3)	0.053 (4)	0.017 (4)	0.039 (4)	0.016 (3)
C10'	0.074 (5)	0.049 (3)	0.053 (4)	0.017 (4)	0.039 (4)	0.016 (3)
C9'	0.065 (4)	0.041 (4)	0.050 (4)	0.014 (3)	0.024 (3)	0.004 (3)
C8'	0.096 (6)	0.028 (3)	0.052 (5)	0.002 (3)	0.021 (4)	−0.007 (3)
C7'	0.082 (7)	0.056 (7)	0.079 (5)	−0.027 (5)	0.015 (5)	0.003 (3)
C6'	0.060 (4)	0.069 (6)	0.089 (7)	−0.006 (4)	0.033 (4)	0.026 (5)
C11	0.0376 (18)	0.0352 (18)	0.0442 (19)	−0.0051 (14)	0.0104 (16)	0.0011 (14)
C12	0.0364 (17)	0.0347 (18)	0.0397 (18)	−0.0016 (15)	0.0100 (15)	0.0035 (14)
C13	0.0426 (18)	0.0365 (18)	0.0412 (17)	0.0005 (15)	0.0174 (16)	0.0062 (15)
C14	0.050 (2)	0.063 (3)	0.060 (2)	0.0082 (19)	0.019 (2)	−0.002 (2)
C15	0.081 (3)	0.065 (3)	0.059 (2)	0.022 (2)	0.034 (2)	0.000 (2)
C16	0.089 (3)	0.056 (2)	0.048 (2)	0.006 (2)	0.025 (2)	−0.0055 (19)
C18	0.052 (2)	0.062 (3)	0.049 (2)	0.0017 (18)	0.0176 (19)	−0.0116 (18)
C19	0.053 (2)	0.040 (2)	0.051 (2)	0.0033 (17)	0.018 (2)	0.0017 (16)
N1	0.057 (2)	0.080 (3)	0.064 (2)	0.0226 (18)	−0.0024 (18)	0.0045 (18)
N17	0.068 (2)	0.072 (2)	0.056 (2)	0.0028 (18)	0.0149 (18)	−0.0192 (17)

### Geometric parameters (Å, °)

**Table:**

Fe1—C8'	2.009 (18)	C9—C10	1.420 (8)
Fe1—C9'	2.018 (15)	C9—H9	0.9300
Fe1—C5	2.018 (3)	C10—H10	0.9300
Fe1—C7	2.026 (10)	C10'—C9'	1.38 (2)
Fe1—C10'	2.026 (18)	C10'—C6'	1.39 (3)
Fe1—C6	2.029 (8)	C10'—H10'	0.9300
Fe1—C1	2.030 (3)	C9'—C8'	1.31 (2)
Fe1—C2	2.035 (3)	C9'—H9'	0.9300
Fe1—C8	2.040 (6)	C8'—C7'	1.45 (3)
Fe1—C3	2.045 (3)	C8'—H8'	0.9300
Fe1—C4	2.049 (3)	C7'—C6'	1.44 (3)
Fe1—C10	2.050 (6)	C7'—H7'	0.9300
C1—C5	1.439 (4)	C6'—H6'	0.9300
C1—C11	1.440 (4)	C11—C12	1.345 (4)
C1—C2	1.440 (4)	C11—H11A	0.9300
C2—C3	1.401 (5)	C12—C19	1.446 (5)
C2—H2	0.9300	C12—C13	1.482 (4)
C3—C4	1.408 (5)	C13—C14	1.379 (4)
C3—H3	0.9300	C13—C18	1.384 (4)
C4—C5	1.411 (4)	C14—C15	1.377 (5)
C4—H4	0.9300	C14—H14A	0.9300
C5—H5	0.9300	C15—C16	1.368 (5)
C6—C7	1.405 (12)	C15—H15A	0.9300
C6—C10	1.416 (10)	C16—N17	1.323 (4)
C6—H6	0.9300	C16—H16A	0.9300
C7—C8	1.417 (11)	C18—N17	1.334 (4)
C7—H7	0.9300	C18—H18A	0.9300
C8—C9	1.411 (8)	C19—N1	1.134 (4)
C8—H8	0.9300		
			
C8'—Fe1—C9'	37.9 (6)	C4—C5—C1	108.9 (3)
C8'—Fe1—C5	117.0 (7)	C4—C5—Fe1	70.89 (19)
C9'—Fe1—C5	112.2 (5)	C1—C5—Fe1	69.61 (18)
C8'—Fe1—C7	52.6 (7)	C4—C5—H5	125.6
C9'—Fe1—C7	67.9 (6)	C1—C5—H5	125.6
C5—Fe1—C7	164.2 (4)	Fe1—C5—H5	125.5
C8'—Fe1—C10'	65.1 (8)	C7—C6—C10	108.2 (8)
C9'—Fe1—C10'	40.0 (7)	C7—C6—Fe1	69.6 (5)
C5—Fe1—C10'	136.0 (7)	C10—C6—Fe1	70.5 (4)
C7—Fe1—C10'	54.7 (6)	C7—C6—H6	125.9
C8'—Fe1—C6	67.8 (7)	C10—C6—H6	125.9
C9'—Fe1—C6	53.4 (6)	Fe1—C6—H6	125.6
C5—Fe1—C6	152.7 (4)	C6—C7—C8	108.8 (8)
C7—Fe1—C6	40.5 (3)	C6—C7—Fe1	69.8 (5)
C10'—Fe1—C6	18.1 (6)	C8—C7—Fe1	70.1 (5)
C8'—Fe1—C1	146.7 (7)	C6—C7—H7	125.6
C9'—Fe1—C1	116.4 (5)	C8—C7—H7	125.6
C5—Fe1—C1	41.65 (12)	Fe1—C7—H7	126.0
C7—Fe1—C1	153.6 (3)	C9—C8—C7	107.0 (7)
C10'—Fe1—C1	110.3 (6)	C9—C8—Fe1	70.2 (3)
C6—Fe1—C1	118.7 (3)	C7—C8—Fe1	69.1 (5)
C8'—Fe1—C2	171.8 (7)	C9—C8—H8	126.5
C9'—Fe1—C2	146.9 (7)	C7—C8—H8	126.5
C5—Fe1—C2	69.11 (13)	Fe1—C8—H8	125.7
C7—Fe1—C2	120.1 (3)	C8—C9—C10	109.0 (6)
C10'—Fe1—C2	114.8 (7)	C8—C9—Fe1	69.4 (3)
C6—Fe1—C2	109.4 (3)	C10—C9—Fe1	69.7 (3)
C1—Fe1—C2	41.49 (12)	C8—C9—H9	125.5
C8'—Fe1—C8	18.9 (5)	C10—C9—H9	125.5
C9'—Fe1—C8	54.5 (5)	Fe1—C9—H9	126.9
C5—Fe1—C8	125.4 (3)	C6—C10—C9	107.1 (6)
C7—Fe1—C8	40.8 (3)	C6—C10—Fe1	68.9 (4)
C10'—Fe1—C8	72.1 (6)	C9—C10—Fe1	69.8 (3)
C6—Fe1—C8	68.6 (4)	C6—C10—H10	126.5
C1—Fe1—C8	163.7 (3)	C9—C10—H10	126.5
C2—Fe1—C8	153.2 (3)	Fe1—C10—H10	126.5
C8'—Fe1—C3	135.2 (7)	C9'—C10'—C6'	109.4 (18)
C9'—Fe1—C3	172.9 (7)	C9'—C10'—Fe1	69.7 (10)
C5—Fe1—C3	68.07 (14)	C6'—C10'—Fe1	71.3 (13)
C7—Fe1—C3	109.8 (3)	C9'—C10'—H10'	125.3
C10'—Fe1—C3	144.5 (8)	C6'—C10'—H10'	125.3
C6—Fe1—C3	129.4 (3)	Fe1—C10'—H10'	125.3
C1—Fe1—C3	68.78 (13)	C8'—C9'—C10'	107.6 (17)
C2—Fe1—C3	40.17 (13)	C8'—C9'—Fe1	70.7 (10)
C8—Fe1—C3	119.2 (3)	C10'—C9'—Fe1	70.3 (9)
C8'—Fe1—C4	112.4 (6)	C8'—C9'—H9'	126.2
C9'—Fe1—C4	135.5 (7)	C10'—C9'—H9'	126.2
C5—Fe1—C4	40.59 (13)	Fe1—C9'—H9'	124.4
C7—Fe1—C4	127.7 (4)	C9'—C8'—C7'	112.8 (19)
C10'—Fe1—C4	175.1 (8)	C9'—C8'—Fe1	71.4 (10)
C6—Fe1—C4	166.2 (4)	C7'—C8'—Fe1	71.1 (14)
C1—Fe1—C4	69.28 (13)	C9'—C8'—H8'	123.6
C2—Fe1—C4	68.28 (14)	C7'—C8'—H8'	123.6
C8—Fe1—C4	106.9 (2)	Fe1—C8'—H8'	125.5
C3—Fe1—C4	40.22 (13)	C6'—C7'—C8'	102 (2)
C8'—Fe1—C10	54.8 (6)	C6'—C7'—Fe1	69.4 (16)
C9'—Fe1—C10	19.7 (5)	C8'—C7'—Fe1	67.2 (13)
C5—Fe1—C10	117.6 (3)	C6'—C7'—H7'	129.1
C7—Fe1—C10	68.2 (4)	C8'—C7'—H7'	129.1
C10'—Fe1—C10	23.7 (6)	Fe1—C7'—H7'	125.9
C6—Fe1—C10	40.6 (3)	C10'—C6'—C7'	108 (2)
C1—Fe1—C10	106.8 (2)	C10'—C6'—Fe1	68.9 (13)
C2—Fe1—C10	128.4 (3)	C7'—C6'—Fe1	69.7 (17)
C8—Fe1—C10	68.6 (3)	C10'—C6'—H6'	125.9
C3—Fe1—C10	166.7 (3)	C7'—C6'—H6'	125.9
C4—Fe1—C10	151.4 (3)	Fe1—C6'—H6'	127.0
C5—C1—C11	123.4 (3)	C12—C11—C1	129.1 (3)
C5—C1—C2	106.0 (3)	C12—C11—H11A	115.4
C11—C1—C2	130.6 (3)	C1—C11—H11A	115.4
C5—C1—Fe1	68.74 (18)	C11—C12—C19	119.4 (3)
C11—C1—Fe1	124.4 (2)	C11—C12—C13	125.9 (3)
C2—C1—Fe1	69.45 (18)	C19—C12—C13	114.6 (3)
C3—C2—C1	108.2 (3)	C14—C13—C18	116.8 (3)
C3—C2—Fe1	70.3 (2)	C14—C13—C12	121.6 (3)
C1—C2—Fe1	69.06 (17)	C18—C13—C12	121.6 (3)
C3—C2—H2	125.9	C15—C14—C13	119.6 (3)
C1—C2—H2	125.9	C15—C14—H14A	120.2
Fe1—C2—H2	126.3	C13—C14—H14A	120.2
C2—C3—C4	109.4 (3)	C16—C15—C14	118.9 (4)
C2—C3—Fe1	69.54 (19)	C16—C15—H15A	120.5
C4—C3—Fe1	70.06 (19)	C14—C15—H15A	120.5
C2—C3—H3	125.3	N17—C16—C15	123.3 (4)
C4—C3—H3	125.3	N17—C16—H16A	118.3
Fe1—C3—H3	126.7	C15—C16—H16A	118.3
C3—C4—C5	107.6 (3)	N17—C18—C13	124.4 (3)
C3—C4—Fe1	69.7 (2)	N17—C18—H18A	117.8
C5—C4—Fe1	68.52 (19)	C13—C18—H18A	117.8
C3—C4—H4	126.2	N1—C19—C12	177.5 (4)
C5—C4—H4	126.2	C16—N17—C18	117.1 (3)
Fe1—C4—H4	127.1		
